# Polimorfismos de los genes del sistema leptina- melanocortina asociados con la obesidad en la población adulta de Barranquilla

**DOI:** 10.7705/biomedica.4827

**Published:** 2020-06-30

**Authors:** Pilar Garavito, María Isabel Mosquera-Heredia, Luis Fang, Fausto Payares, Marta Ruiz, Isis Arias, Rafael Tuesca, Édgar Navarro, Carlos Silvera-Redondo

**Affiliations:** 1 Grupo de Investigación Genética y Medicina Molecular, Departamento de Medicina, Universidad del Norte, Barranquilla, Colombia Grupo de Investigación Genética y Medicina Molecular Departamento de Medicina, Universidad del Norte Barranquilla Colombia; 2 Grupo de Investigación Proyecto UNI, Departamento de Salud Pública, Universidad del Norte,Barranquilla, Colombia Grupo de Investigación Proyecto UNI Departamento de Salud Pública, Universidad del Norte,Barranquilla Colombia

**Keywords:** obesidad/genética, polimorfismo genético., Obesity/genetics, polymorphism, genetic.

## Abstract

**Introducción.:**

La obesidad se considera un grave problema de salud pública y por ello se hacen esfuerzos en la búsqueda de genes como el *LEP,* el *LEPR* y el *MC4R* del sistema leptina-melanocortina, el cual opera en la regulación neuroendocrina de la ingestión y el equilibrio energético e influye en la patogenia de la enfermedad. Los resultados contradictorios en torno a la asociación de estos genes con la obesidad plantean la necesidad de nuevas investigaciones.

**Objetivo.:**

Analizar los polimorfismos rs2167270 del gen *LEP,* rs1137101 del gen *LEPR* y rs17782313 del gen *MC4R* asociados con la obesidad y sus variables clínicas y bioquímicas en una muestra de pacientes adultos de Barranquilla.

**Materiales y métodos.:**

Se estudiaron 111 personas obesas y 155 no obesas como controles. Los polimorfismos se determinaron mediante reacción en cadena de la polimerasa (PCR) en tiempo real. Se tomaron las medidas antropométricas, se evaluó la presión arterial y se hicieron pruebas bioquímicas.

**Resultados.:**

No se encontraron diferencias estadísticas en la frecuencia alélica y genotípica de los polimorfismos en los grupos estudiados. En cuanto a las variables clínicas y bioquímicas, el genotipo CC del polimorfismo rs17782313 del gen *MC4R*, se asoció con un aumento de la presión arterial sistólica y, el alelo T y su genotipo homocigoto, con una disminución del colesterol HDL en los obesos. No se evidenció ningún efecto de los otros polimorfismos en estas variables.

**Conclusiones.:**

Los polimorfismos rs2167270 del gen *LEP,* rs1137101 del gen *LEPR* y rs17782313 del gen *MC4R*, no se asociaron con obesidad en la población analizada. Se encontró que el polimorfismo rs17782313 del gen *MC4R* influyó en el aumento de la presión arterial sistólica y la disminución del colesterol HDL en las personas obesas.

## Abstract (en)

**Introduction:**

Obesity is considered a serious public health problem. Efforts have been directed to search for candidate genes such as *LEP, LEPR,* and *MC4R* involved in the leptin- melanocortin system. The neuroendocrine regulation of these genes on energy intake and balance influences the pathogenesis of this disease. Contradictory results regarding the association of these genes with obesity raise the need for new research.

**Objective:**

To analyze the association between obesity and *LEP* rs2167270, *LEPR* rs1137101, and *MC4R* rs17782313 polymorphisms and the clinical and biochemical variables in obese adults from Barranquilla, Colombia.

**Materials and methods:**

We analyzed 111 obese adults and 155 non-obese individuals as controls. The polymorphisms were determined by real-time PCR. Besides, anthropometric measures, blood pressure, and biochemical tests were evaluated.

**Results:**

No statistical differences were found in allele and genotype frequencies of gene polymorphisms between groups. The CC genotype of *MC4R* rs17782313 polymorphism was associated with increased systolic blood pressure and T allele and TT genotype, with decreased HDL cholesterol in obese adults. The effect of the other polymorphisms on these variables was not evidenced.

**Conclusions:**

*LEP* rs2167270, *LEPR* rs1137101, and *MC4R* rs17782313 polymorphisms were not associated with obesity in the population under study. *MC4R* rs17782313 polymorphisms were associated with an increase in systolic blood pressure and a decrease in HDL cholesterol.

La obesidad se considera un grave problema de salud pública a nivel mundial, ya que constituye el principal tipo de malnutrición del adulto. Según la Organización Mundial de la Salud (OMS), en el 2016, más de 1.900 millones de adultos tenían exceso de peso, lo que representa el 39 % de la población, y el 13 % de ellos se consideraban obesos ^1^.

El gran impacto de la obesidad en la morbilidad y la calidad de vida de los individuos se debe a que constituye un factor de riesgo de diabetes, cardiopatías isquémicas, enfermedades del aparato locomotor y algunos tipos de cáncer ^1^. Por lo tanto, la comprensión de su etiología y de la forma de regular el peso corporal tiene gran importancia científica, sanitaria y económica.

Un elemento clave esencial para comprender la etiología y la patogenia de la obesidad es el papel del sistema leptina-melanocortinas en el control del almacenamiento de la grasa corporal mediante la regulación coordinada de la conducta alimentaria, el metabolismo, las reacciones neuroendocrinas, el sistema nervioso autónomo y el balance de energía del cuerpo ^2^.

El gen *LEP* está situado en 7q32.1, mide 20 kb y está constituido por tres exones y dos intrones; codifica para la leptina, una hormona proteica constituida por 167 aminoácidos con peso molecular de 16 kDa, en tanto que su receptor, codificado por el gen *LEPR*, está constituido por 24 exones y se localiza en 1p31.3. Por su parte, el gen *MC4R* se ubica en 18q22, solo tiene un exon y codifica para el receptor 4 de melanocortina.

La leptina es secretada fundamentalmente por el tejido adiposo blanco. Sus concentraciones circulantes son proporcionales al contenido de la grasa corporal ^3^. Esta hormona atraviesa la barrera hematoencefálica e interactúa con su receptor específico en el núcleo arcuato del hipotálamo y actúa como una señal indicadora de las reservas energéticas. Las POMC/ CART y las AGRP/NPY son las poblaciones neuronales del núcleo arcuato que expresan el receptor de la leptina en forma importante. Las primeras, las POMC/CART, conducen señales anorexigénicas mediante los derivados de la proopiomelanocortina (POMC), en tanto que las AGRP/NPY conducen señales orexigénicas mediante el neuropéptido Y (NPY) y y la proteína relacionada con agouti (*Agouti-related protein*, *AgRP)*. La ausencia de leptina o la reducción de su concentración provocan una mayor ingestión alimentaria debido a la expresión de AGRP/NPY. En el caso contrario, se promueve la expresión de la POMC, la cual se escinde después de su traducción en péptidos llamados melanocortinas, que actuarían como ligandos endógenos del receptor 4 de melanocortina (MC4R), disminuyendo el apetito ^4^.

Cuando se reduce la capacidad de la leptina para regular el apetito y el aumento de peso, se desarrolla una resistencia contra ella que puede conducir a la obesidad. Algunos mecanismos implicados en dicha resistencia son los defectos en el transporte de la leptina a través de la barrera hematoencefálica, los defectos en la señalización del receptor de la leptina y las alteraciones en las vías nerviosas involucradas en la regulación de la homeostasis energética ^5^.

Se ha demostrado que algunos polimorfismos de un solo nucleótido (SNP) en los genes *LEP*, *LEPR* y *MC4R*, se relacionan con el fenotipo y los marcadores metabólicos asociados con la obesidad. Se ha sugerido que el polimorfismo rs2167270 del gen *LEP* podría estar asociado con la variación en el aumento de la leptinemia y la propensión a la obesidad ^6^. Asimismo, el SNP *rs1137101* del gen *LEPR* también se ha relacionado con un aumento en el índice de masa corporal (IMC), hiperleptinemia y predisposición a la resistencia a la leptina ^7^.

Por su parte, los metaanálisis de estudios de asociación en todo el genoma (*Genome-Wide Association Study*, GWAS) en caucásicos, revelaron que la variante rs17782313 del gen *MC4R* tiene una fuerte asociación con valores elevados del IMC ^8^ y con la aparición temprana de obesidad grave (9). Estas asociaciones se han replicado en múltiples poblaciones, incluidos niños, adolescentes y adultos. Sin embargo, se han reportado resultados contradictorios alrededor del mundo, lo cual indica la necesidad de adelantar nuevas investigaciones al respecto.

El objetivo del presente estudio fue analizar los polimorfismos rs2167270 del gen *LEP,* rs1137101 del gen *LEPR* y rs17782313 del gen *MC4R* asociados con la obesidad en una muestra de adultos de Barranquilla.

## Materiales y métodos

### Población y muestra

Se hizo un estudio descriptivo transversal con análisis de casos y controles, lo que implicó hacer un muestreo poblacional en Barranquilla. Se compararon dos grupos en el rango de edad entre los 20 y los 69 años: los casos eran individuos con obesidad y un IMC de 30 kg/m^2^ o mayor, y los sujetos de control tenían un IMC entre 18,5 y 24,9 kg/m^2^.

La población analizada era una muestra de los participantes en el estudio “Salud global: estrategia de investigación aplicada para el estudio y la intervención del síndrome metabólico en Barranquilla”. El proceso de selección y la técnica de recolección de datos se describen en la publicación de Giraldo-Castrillón, *et al.*^10^.

Para estimar el tamaño de la muestra, se utilizó un porcentaje de 61 % en los polimorfismos de los sistemas genéticos bajo estudio, tomando como referencia la frecuencia del polimorfismo del gen *LEP* (A19G) ^11^, un nivel de confianza del 95 %, un poder del 80 % y una relación entre casos y controles de 1:1, lo que resultó en 116 participantes en el grupo de los casos y 116 en el de los controles.

Los criterios de exclusión incluyeron embarazo, hipotiroidismo, síndrome de Cushing, hipogonadismo o síndromes monogénicos relacionados con obesidad, así como estar en tratamiento para diabetes o dislipidemia.

Todos los participantes firmaron un consentimiento informado y el estudio se ajustó a los principios éticos de la investigación en seres humanos consignados en la Declaración de Helsinki y la Resolución 8430 de 1993 del Ministerio de Salud de Colombia. Además, el estudio contó con la aprobación del Comité de Ética de la Universidad del Norte.

### Parámetros clínicos y bioquímicos

Los parámetros antropométricos de peso, talla, cintura y cadera se tomaron siguiendo los protocolos estandarizados. El IMC se calculó con la fórmula de peso (kg)/talla (m^2^). Se clasificó como individuos con sobrepeso a aquellos con un IMC entre 25 y 29,9 kg/m^2^, y con obesidad, a quienes tenían un IMC mayor o igual a 30 kg/m^2^. Se consideró normal una circunferencia de cintura de menos de 88 centímetros en las mujeres y de menos de 102 centímetros en los hombres ^12^. Además, la presión arterial sistólica y la diastólica se midieron en dos momentos diferentes, siguiendo el protocolo de la OMS ^13^. Los puntos de corte se tomaron del *Eighth Joint National Committee* (JNC 8) para la prevención, detección, evaluación y tratamiento de la hipertensión ^14^.

A los individuos participantes se les extrajeron 10 ml de sangre total mediante venopunción convencional después de 12 horas de ayuno; 5 ml se recolectaron en un tubo con EDTA para los estudios genéticos y 5 ml en un tubo seco para los estudios bioquímicos utilizando el sistema Vacutainer™. El colesterol total, los triglicéridos y la glucemia se determinaron con métodos enzimáticos fotocolorimétricos comerciales (Biosystems); el cHDL se separó al precipitar selectivamente las lipoproteínas de baja densidad (*low density lipoprotein,* LDL) y de muy baja densidad (*very low density lipoprotein,* VLDL) agregando ácido fosfotúngstico en presencia de iones de magnesio. El cLDL se calculó de forma indirecta empleando la ecuación de Friedewald ^15^. Los puntos de corte utilizados para la clasificación de la dislipidemia se determinaron a partir de las guías del *Adult Treatment Panel III* (ATP III) ^16^.

La insulina se determinó mediante quimioluminiscencia (Siemens Medical) y la hemoglobina glucosilada por colorimetria por afinidad al boronato (NycoCard Axis-Shield™) siguiendo las instrucciones de la casa comercial.

### Extracción del ADN genómico y genotipificación

El ADN genómico se obtuvo usando el estuche comercial UltraClean BloodTM (MO BIO Laboratories, Inc.), siguiendo las instrucciones recomendadas por el fabricante. La cuantificación del ADN se llevó a cabo en un espectrofotómetro NanoDrop 2000™ que, además de los valores de concentración, también aporta los valores de relación 260/280 (ADN/ proteínas) y 260/230 (ADN y solventes orgánicos) e información sobre la pureza del producto. Se diluyeron todas las muestras de ADN hasta llevarlas a una concentración de 20 ng/μl y se almacenaron a -20 ºC.

Los polimorfismos rs2167270 del gen *LEP,* rs1137101 del gen *LEPR* y rs17782313 del gen *MC4R* se determinaron mediante PCR en tiempo real con el método de discriminación alélica TaqMan PRISM 7500 Real-Time PCR Systems™ (Applied Biosystems) con los ensayos ID C_15966471_20, ID C_8722581_10 y ID C_32667060_10, respectivamente.

La mezcla de amplificación para un volumen final de reacción de 25 μl contenía 20 ng de ADN genómico, 12,5 μl de TaqMan Universal PCR Master Mix™, mezcla que contiene polimerasa AmpliTaq Gold DNA™, AmpErase Uracil N-glicosilasa (UNG), desoxinucleótidos (dNTP) con *desoxiuridina 5*’-*trifosfato* (dUTP), referencia pasiva (ROX) y solución tampón, así como 1,25 μl de 20X de mezcla para cada uno de los ensayos de genotipificación descritos anteriormente y específica para cada polimorfismo, con 18 μM de cada cebador y 4 μM de cada sonda (VIC/FAM).

Todas las pruebas se hicieron siguiendo un mismo protocolo de amplificación y detección que se iniciaba con un ciclo a 50 ºC durante 2 minutos, un ciclo a 95 ºC durante 10 minutos, 40 ciclos a 95 ºC durante 15 segundos cada uno y un ciclo a 60 ºC durante 90 segundos.

Por último, los genotipos se determinaron a partir del resultado de los productos de amplificación observados como curvas de amplificación reconocidas a partir de la marcación para cada sonda (VIC/FAM).

### Análisis estadístico

Para el análisis estadístico, se estimaron las frecuencias alélicas y genotípicas de los SNP estudiados en ambos grupos. Las frecuencias alélicas se usaron para estimar el equilibrio genético de Hardy-Weinberg. El análisis de asociación genética se estimó con el programa Arlequin, versión 3.5.

El análisis de asociación de genotipos se hizo mediante la prueba de ji al cuadrado de Pearson con corrección de Bonferroni para el ajuste del valor de p en el programa estadístico SPSS™, versión 20 (IBM Corp., USA). También, se estimó el riesgo de obesidad asociado con cada genotipo y el alelo mediante el cálculo de la razón de momios (*odds ratio,* OR) y sus correspondientes intervalos de confianza del 95 % usando modelos de regresión logística ajustados por sexo.

Las variables numéricas se expresaron como medianas y rangos intercuartílicos (RIC). Estas variables se compararon en los dos grupos de estudio y entre los polimorfismos mediante las pruebas estadísticas U de Mann-Whitney y Kruskal-Wallis, respectivamente, debido a que no cumplieron con el supuesto de normalidad.

El contraste de las variables categóricas se hizo mediante la prueba de ji al cuadrado de Pearson. A estos datos se les determinó la corrección de Bonferroni del valor de p. La significación estadística se estableció en p<0,05.

## Resultados

Se incluyeron y compararon dos grupos en el rango de edad entre los 20 y los 69 años: fueron 111 casos con obesidad y un IMC de 30 kg/m^2^ o mayor, y 155 de control con un IMC entre 18,5 y 24,9 kg/m^2^.

Estas cantidades corresponden a una disminución del 4,3 % (5 sujetos) en el número de casos y a un incremento de 39 (33,6 %) en el de controles, para evitar la disminución del poder del estudio. Los casos que no participaron abandonaron el estudio por temor a la venopunción y por no haber completado las pruebas bioquímicas.

La edad promedio fue de 40,8 ± 12,3 años entre los casos y de 35 ± 12,7 años entre los controles. En ambos grupos predominó el sexo femenino, con porcentajes del 66,1 y 53,5 %, respectivamente.

En el cuadro 1 se presentan las características clínicas y bioquímicas de la población estudiada, así como las diferencias encontradas al comparar los grupos. La edad, las medidas antropométricas, la glucemia en ayunas, los triglicéridos, la HbA1C y el riesgo cardiovascular global, fueron significativamente mayores en el grupo de los casos.

**Cuadro 1:** Características clínicas y bioquímicas de la población analizada

	Casos		Control		p	OR	IC_95%_
		(n=111) n (%)		(n=155) n (%)			
Sexo							
Masculino	41	(36,9)	72	(46,5)		1	---
Femenino	70	(63,1)	83	(53,5)	0,155^†^	1,48	0,9-2,4
Edad (años)							
Mediana [RIC]	40	(19)	32	[20]	0,00^*¥^		
Riesgo cardiovascular							
Muy bajo	11	(9,9)	46	(29,7)		1	---
Bajo	21	(18,9)	28	(18,1)		3,42	1,4-8,2
Alto	79	(71,2)	81	(52,3)	0,00^*†^	5,79	2,3-14,5
Hipertensión							
Normal <120/<80 mm Hg	58	(52,3)	97	(62,6)		1	---
Prehipertenso 120-139/80-89 mm Hg	34	(30,6)	41	(26,5)		1,3	0,7-2,4
HTA-1 140-159/90-99 mm Hg	13	(11,7)	15	(9,7)		1,6	0,7-3,7
HTA-2 >160/>100 mm Hg	6	(5,4)	2	(1,3)	0,14^†^	5	0,9-26
IMC (kg/m^2^)							
Mediana (RIC)	33,04	(4,76)	22,6	(2,72)	0,00^*¥^		
Perímetro braquial (cm)							
Mediana (RIC)	37,5	(5,5)	29	(5)	0,00^*¥^		
Perímetro de cintura (cm)							
Mediana (RIC)	105	(14)	81,6	(12,2)	0,00^*¥^		
Perímetro de cadera (cm)							
Mediana (RIC)	112	(12,5)	90	(11,2)	0,00^*¥^		
Índice cintura-cadera							
Mediana (RIC)	0,94	(0,08)	0,92	(0,07)	0,004^*¥^		
Porcentaje de grasa corporal							
Mediana (RIC)	31,4	(18)	25,6	(15)	0,003^*¥^		
Glucemia en ayunas (mg/dl)							
Mediana (RIC)	85	(15,5)	83	(15)	0,046^*¥^		
Colesterol total (mg/dl)							
Mediana (RIC)	197	(57)	189	(62)	0,492		
Mediana (RIC)							
cLDL (mg/dl)	41	(16)	41	(15)	0,887		
Mediana (RIC)							
VLDL (mg/dl)	125	(49,8)	117,8	(48,4)	0,788		
VLDL (mg/dl)							
Mediana (RIQ)	28,4	(22,6)	24,9	(19,05)	0,071		
Triglicéridos (mg/dl)							
Mediana (RIC)	144	(120)	123	(94)	0,043^*¥^		
Insulina (mUL)							
Mediana (RIC)	4,65	(10,2)	4,15	(7,29)	0,107		
Hb “glucosilada” (%)							
Mediana (RIC)	5,7	5,7 (0,9)	5,4	(1)	0,002^*¥^		

IMC: indice de masa corporal; cHDL cLDL y VLDL: lipoproteinas de alta, baja y muy baja densidad,

respectivamente; T_SIS_MED y T_DIAS_MED: tension arterial sistolica y diastolica media, respectivamente

* p<0,05

RIC: rango intercuartilico

† prueba de ji al cuadrado de Pearson

\ prueba U de Mann-Whitney

 Las frecuencias alelicas y genotipicas de los polimorfismos estudiados, se presentan en el cuadro 2. Se observo que estas frecuencias se mantuvieron en equilibrio de Hardy-Weinberg en el grupo de control, excepto para el polimorfismo del gen *MC4R*. Tambien, se presenta en este cuadro un análisis de asociacion de estas frecuencias con la obesidad, el cual no evidencio diferencias estadisticas entre los grupos para ninguno de los SNP estudiados. 

**Cuadro 2:** Frecuencias genotipicas y alelicas de los polimorfismos rs2167270 del gen *LEP*, rs1137101 del gen *LEPR* y rs17782313 del gen *MC4R*

	Casos			Controles			p^¥^	OR	IC_95%_
	(n=111) n	%	H-W^†^	n=155	%	H-W^†^			
LEP									
rs2167270									
AA	21	18,9		27	17,40				
AG	42	37,8		71	45,80		0,42	0,9	0,4-1,8
GG	48	43,2	0,044*	57	36,80	0,06		0,73	0,4-1,2
G	138	62,2		185	59,7		0,625	1	---
A	84	37,8		125	40,30				
LEPR									
rs1137101									
AA	38	34,2		50	32,30				
AG	49	44,1		70	45,20		0,943	1	---
GG	24	21,6	0,33	35	22,60	0,26		0,89	0,5-1,5
A	125	56,3		170	54,80		0,805	0,91	0,4-1,7
G	97	43,7		140	45,20				
MC4R									
rs17782313									
TT	83	74,8		116	74,80			1	---
CT	25	22,5		32	20,60		0,715	0,58	0,1-2,3
CC	3	2,7	0,44	7	4,50	0,047*		1,07	0,5-1,9
T	191	86		264	85,20		0,875		
C	31	14		46	14,80				

† test de equilibrio de Hardy-Weinberg mejorado con 100.000 pasos de la cadena Markov

￥razon de verosimilitud de ji al cuadrado; OR (IC_95%_): modelos de regresion logistica ajustados por sexo

**Figura 1:**
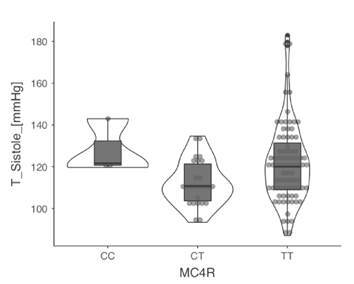
Asociación del polimorfismo rs17782313 del gen *MC4R* con la tensión arterial sistólica promedio en el grupo de adultos obesos En los individuos obesos con genotipo TT hubo una disminución de las concentraciones séricas de HDL (mediana: 41; RIC: 15), a diferencia de los individuos con genotipos CC (mediana: 43; RIC: 14,7) y CT (mediana: 49; RIC: 18; p: 0,048).

Además, se analizó la asociación de los polimorfismos con las medidas antropométricas, la presión arterial y las pruebas bioquímicas en los dos grupos de estudio y se encontró que el genotipo CC del polimorfismo rs17782313 del gen *MC4R* se asoció con un aumento de la presión arterial sistolica y, el alelo T y su genotipo homocigoto, con una disminución del cHDL, pero solo en el grupo de obesos. No se evidenci asociacion de los otros polimorfismos con las variables estudiadas cuadro 3, figuras 1 y 2.

**Cuadro 3:** Asociación de los polimorfismos rs2167270 del gen *LEP*, rs1137101 del gen *LEPR* y rs17782313 del gen *MC4R*, con las variables clínicas y bioquímicas

**Grupo de casos**												
	***LEP***				***LEPR***				***MC4R***			
	**AA (n=21) Mediana (RIC)**	**AG (n=42) Mediana (RIC)**	**GG (n=48) Mediana (RIC)**	**Valor p^¥^**	**AA (n=38) Mediana (RIC)**	**AG (n=49) Mediana (RIC)**	**GG (n=24) Mediana (RIC)**	**Valor p^¥^**	**CC (n=3) Media (DE)**	**CT (n=25) Mediana (RIC)**	**TT (n=83) Mediana (RIC)**	**p^¥^**
IMC [kg/m2]	32,2 [3,4]	33,1 [6,3]	33,9 [4,6]	0,655	34,01 [5,3]	32,6 [4,1]	32,8 [6,4]	0,53	32,8 [1,5]	33,4 [3,7]	33,04 [5,4]	0,94
Índice cintura/cadera	0,92 [0,06]	0,95 [0,07]	0,94 [0,07]	0,103	0,95 [0,07]	0,94 [0,07]	0,94 [0,1]	0,812	0,96 [0,07]	0,92 [0,08]	0,95 [0,06]	0,147
T_SIS_MED (mm Hg)	113,3 [21,5]	117,6 [25,5]	119,8 [20]	0,853	118,6 [14,5]	119,6 [20]	115,5 [35,1]	0,967	128,1 [12,9]	110,6 [18,3]	120 [24,6]	0,025*
T_DIAS_MED (mm Hg)	71,3 [18,6]	72 [16,7]	75,5 [14]	0,724	76,5 [16,8]	72,6 [13,5]	69,5 [22]	0,832	82 [9,5]	69,6 [11,1]	75,6 [15,6]	0,077
% grasa corporal	28,1 [15,7]	37,6 [16,5]	30,3 [17,6]	0,089	32,1 [16,6]	29,7 [18]	35,6 [19,5]	0,845	24,5 [8,9]	29,7 [19,5]	31,7 [16]	0,409
Glucemia (mg/dl)	82 [24,7]	87 [16]	84,5 [14,5]	0,57	83,8 [11,5]	83 [20,5]	88,5 [22,5]	0,077	87,5 [8,2]	82 [16,5]	86 [16]	0,234
Colesterol (mg/dl)	184 [34]	204 [74,2]	196 [57,7]	0,485	184,5 [55,5]	195 [66,5]	205,5 [51,7]	0,094	175,3 [19,08]	198 [36,5]	197 [69]	0,468
cHDL (mg/dl)	42 [27]	41 [14,2]	42 [16]	0,524	41 [13,7]	42 [17]	41,5 [14]	0,64	43 [14,7]	49 [18]	41 [15]	0,048*
cLDL (mg/dl)	112,8 [39,6]	127,3 [51,5]	127,1 [53,4]	0,393	113,1 [56,5]	124,8 [50,4]	133 [39,4]	0,252	103,4 [22,1]	127 [47,8]	124 [57,2]	0,554
VLDL (mg/dl)	32,6 [23]	27,2 [27,1]	25 [16,5]	0,728	25,2 [19,8]	28,4 [24,9]	29,3 [16,8]	0,951	28,9 [11,5]	28,4 [19,2]	28,8 [25,8]	0,612
Triglicéridos (mg/dl)	161 [114]	136 [132]	131 [91,7]	0,616	123 [122,3]	146 [116]	146,5 [112,5]	0,861	154,9 [59,4]	145 [96,5]	141 [129]	0,793
Insulina (mULO)	5,02 [9,4]	3,98 [9,1]	6,64 [11,5]	0,742	4,76 [11,7]	5,52 [9,95]	3,60 [11,07]	0,963	15,3 [13,4]	7,9 [11,5]	3,96 [9,2]	0,157
HbA1c (%)	5,9 [0,5]	5,8 [1,175]	5,6 [0,9]	0,085	5,75 [1,1]	5,7 [0,7]	5,7 [1,1]	0,66	5,5 [0,28]	5,8 [0,55]	5,7 [1]	0,623
Grupo de control												
	*LEP*				*LEPR*				*MC4R*			
	AA (n=27) Mediana (RIC)	AG (n=71) Mediana (RIC)	GG (n=57) Mediana (RIC)	Valor p^¥^	AA (n=50) Mediana (RIC)	AG (n=70) Mediana (RIC)	GG (n=35) Mediana (RIC)	Valor p^¥^	CC (n=7) Mediana (RIC)	CT (n=32) Mediana (RIC)	TT (n=116) Mediana (RIC)	p^¥^
IMC	22,5 (2,4)	22,2 (3,1)	22,9 (2,4)	0,153	22,9 (2,9)	22,6 (2,6)	22,07 (2,2)	0,151	24,06 (2,01)	22,3 (2,9)	22,6 (2,5)	0,068
Índice cintura/cadera	0,92 (0,08)	0,92 (0,08)	0,92 (0,07)	0,967	0,90 (0,08)	0,92 (0,08)	0,93 (0,05)	0,356	0,94 (0,10)	0,90 (0,07)	0,92 (0,07)	0,591
T_SIS_MED	108,3 (26,3)	111,6 (16,6)	114,6 (19,8)	0,304	115 (16,1)	110,6 (22,5)	111,6 (23)	0,682	106,3 (37)	115,5 (25)	112 (16,2)	0,738
T_DIAS_MED	71,6 (12,6)	72,3 (16,3)	72 (12,5)	0,893	72,5 (14,6)	71,5 (13,5)	72,3 (16,3)	0,58	68,3 (28,3)	73,8 (14,7)	71,8 (13)	0,303
% grasa corporal	26,8 (13,3)	25,3 (16,9)	26,1 (14,1)	0,953	25,4 (15,3)	25,5 (16,8)	28,4 (14,3)	0,481	25,3 (15)	23,05 (19,2)	26,4 (14,1)	0,685
Glucemia (mg/dl)	82 (15)	84 (15)	82 (16)	0,832	84,5 (12)	82 (16,7)	84 (16)	0,936	87 (18)	84,5 (15,7)	82 (15)	0,263
Colesterol (mg/dl)	183 (49)	186 (56)	200 (65,5)	0,547	182 (64,2)	187,5 (53,2)	194 (65)	0,802	173 (75)	175,5 (64,5)	192 (58,5)	0,419
cHDL (mg/dl)	39 (21)	40 (14)	44 (15,5)	0,561	43 (19,2)	40,5 (15)	40 (13)	0,87	47 (16)	41,5 (17,2)	40,5 (15)	0,716
cLDL (mg/dl)	112,4 (36,5)	115,6 (45)	120 (65,4)	0,776	113,7 (48,3)	120 (46,3)	118 (64,8)	0,756	114,8 (38,2)	106,4 (52,3)	120 (52,8)	0,391
VLDL (mg/dl)	23,4 (15,7)	24,1 (18,8)	26 (23)	0,6	26 (19,2)	23,6 (18,9)	27 (21,6)	0,425	22,2 (10,8)	26,4 (16,7)	24,6 (20,4)	0,871
Triglicéridos (mg/dl)	123 (79)	122 (97)	126 (88,5)	0,969	126,5 (102)	114,5 (93,2)	134 (97)	0,421	111 (54)	131 (70)	121,5 (102)	0,86
Insulina (mUL)	4,97 (7,1)	3,58 (7,08)	3,91 (7,6)	0,398	4,3 (7,3)	4,55 (6,3)	3,51 (9,6)	0,753	5,56 (8,1)	2,5 (6,8)	4,2 (7,4)	0,897
HbA1c (mg/dl)	5,6 (1,1)	5,3 (0,8)	5,5 (1,05)	0,081	5,4 (0,95)	5,3 (1,1)	5,4 (1,1)	0,828	5,1 (1,3)	5,2 (0,9)	5,4 (0,97)	0,432

IMC: índice de masa corporal; cHDL cLDL y VLDL: lipoproteínas de alta, baja y muy baja densidad, respectivamente; T_SIS_MED y T_DIAS_MED: tensión arterial sistólica y diastólica media, respectivamente

* p<0,05

^¥^ prueba U de Mann-Whitney

En los individuos obesos con genotipo CC, hubo un aumento de la tensión arterial sistólica promedio expresado como mediana de 128 (RIC: 12,9), a diferencia de los individuos con genotipos heterocigoto CT (mediana: 110,6; RIC: 18,3) y homocigoto TT (mediana: 120; RIC: 24,6; p: 0,025).

**Figura 2:**
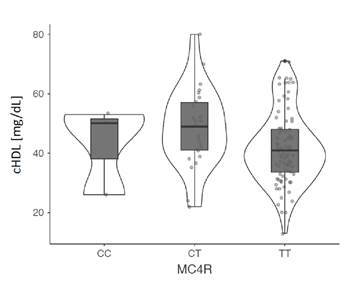
Asociación del polimorfismo rs17782313 del gen *MC4R* con las concentraciones séricas de HDL en el grupo de adultos obesos

## Discussion

Numerosos estudios se han enfocado en evaluar la presencia de algunos SNP en los genes de las proteínas que participan en el sistema leptina- melanocortina, para dilucidar la propensión genética a la obesidad con base en el papel de este sistema en la mantención del equilibrio entre el estímulo y la inhibición del apetito en función del gasto energético para controlar el peso corporal ^17^. En este sentido, en todo el mundo se han obtenido resultados contradictorios, ya que la asociación de estos polimorfismos con el IMC se ve influenciada por la etnia, la ubicación geográfica y los factores medioambientales; de ahí, la necesidad de estudiar la asociación de los polimorfismos rs2167270 del gen *LEP,* rs1137101 del gen *LEPR* y rs17782313 del gen *MC4R* con la obesidad en una población colombiana.

Entre las variantes comunes del gen *LEP*, el polimorfismo rs2167270 ha sido uno de los más frecuentemente asociados con la obesidad. En el *Family Heart Study*, por ejemplo, un haplotipo común (49 %) que incluía este polimorfismo se asoció con un aumento del IMC en población de Estados Unidos ^18^. Asimismo, en un estudio de sujetos del *Coronary Artery Risk Development in Young Adults Study* (CARDIA), este polimorfismo mostró una asociación consistente con tres medidas antropométricas: el peso, el IMC y el perímetro de cintura de sujetos caucásicos ^19^. Además, Shruti, *et al*., encontraron una tendencia al aumento de los niveles del IMC y de leptina con cada adición del alelo A de la variante exónica rs2167270 en una población del sur de India ^20^. Sin embargo, en el presente estudio la frecuencia de este alelo A fue de 37,8 % en el grupo de casos y de 40,3 % en el grupo control, es decir, no tuvo significación estadística.

A pesar de que se ha reportado una fuerte asociación de este polimorfismo con la obesidad, no es posible sugerir una influencia directa de dicha variante en la expresión de la leptina y su función en la fisiopatología de la obesidad, ya que se encuentra dentro del primer exón no traducido del gen. Sin embargo, se puede sugerir que al estar en desequilibrio de ligamiento con el polimorfismo del promotor (rs7799039), rs2167270 puede tener un efecto sobre la transcripción del gen *LEP*^21^*.*

En cuanto al polimorfismo rs1137101 del gen *LEPR*, se ha reportado que su genotipo GG se asoció con un menor riesgo de obesidad (OR=0,26; IC95% 0,08-0,79) (p=0,018), pues redujo el IMC en 2,44 kg/m^2^ en individuos del sur de Chile ^22^. Asimismo, Chavarría-Ávila, *et al*., han señalado que el alelo G podría ser un marcador genético asociado con una menor acumulación de grasa corporal en la población del oeste mexicano ^23^. En este sentido, la frecuencia del alelo G en nuestra población fue predominante (45,2 %) en el grupo con peso normal (p>0,05), lo cual es similar a lo encontrado en poblaciones de Chile y México. Por su parte, el genotipo AA se asoció significativamente con la obesidad en voluntarios de Túnez (OR=1,41; IC95% 1,035-1,85) (p=0,045) ^24^.

En el presente estudio, la frecuencia de este genotipo predominó en el grupo de obesos (p>0,05). Por el contrario, De Oliveira, *et al.*, reportaron una fuerte asociación del genotipo GG con el exceso de peso en población brasilera (OR=2,14, IC95% 1,01-4,52) (p=0,047) ^25^, al igual que Mărginean, *et al*., quienes concluyeron que los sujetos portadores de los genotipos GG o AG tenían 3,06 veces más riesgo de desarrollar obesidad en comparación con los portadores de AA (OR=3,06; IC95% 1,70-5,51) (p=0,0001) ^26^.

Estas discrepancias de los polimorfismos de *LEP* y *LEPR* también se evidenciaron en un metaanálisis del 2015 de Ghalandari, *et al*. ^27^. De los diecisiete artículos revisados, nueve informaban sobre una asociación significativa, o los consideraban como posibles factores de riesgo; sin embargo, no se encontró esta relación en los ocho estudios restantes, que incluían, entre otras, poblaciones de Brasil y México con resultados similares a los del presente estudio.

Lo mismo sucede con el polimorfismo rs17782313 del gen *MC4R*, el cual se ha asociado con un elevado IMC (^8^, ^28^, ^(^^29^) y con la aparición temprana de obesidad grave ^9^. Se ha sugerido que los portadores del genotipo TT de este polimorfismo tienen un nivel notablemente mayor de hipermetilación en el promotor de *MC4R* en comparación con los portadores de los genotipos CT y CC, lo que puede contribuir a regular la expresión de *MC4R* y, por consiguiente, al desarrollo de la obesidad ^30^. Sin embargo, en otros estudios no se ha podido demostrar la asociación de este polimorfismo con la obesidad, como es el caso de las investigaciones en población polaca y africana ^8^^,^^31^. En este sentido, un hallazgo notable del presente estudio es que el genotipo TT fue igual en los casos y los controles.

En resumen, el análisis genético del presente estudio, comparado con los de otras poblaciones, evidenció la falta de asociación de los polimorfismos con la obesidad, resultados que se explicarían porque las poblaciones están expuestas a diferentes factores medioambientales o de estilo de vida, y ello es fundamental para determinar la influencia de cada polimorfismo en la obesidad. Se hace necesario analizar la interacción entre polimorfismo y ambiente para aclarar la contribución de estas variantes genéticas al desarrollo de la obesidad.

Así como en este estudio, otros investigadores han evaluado la influencia de estos polimorfismos en las variables clínicas y bioquímicas relacionadas con la obesidad. En cuanto al perfil de lipoproteínas, se pudo demostrar que los portadores del alelo G del polimorfismo rs1137101 del gen *LEPR* tenían niveles más bajos de triglicéridos y lipoproteínas de muy baja densidad (VLDL) en el sur de Chile ^22^, y niveles más elevados de colesterol total, pero más reducidos de cHDL en sujetos de Túnez ^32^. Por su parte, el alelo C del polimorfismo rs17782313 del gen *MC4R* se ha asociado con una disminución de la lipoproteína antiaterogénica en mujeres italianas con diabetes gestacional y mujeres sanas de China ^33^^,^^34^, resultado similar al reportado en escolares mexicanos (OR=2,99, IC95% 1,93-4,64) (p<0,0001). Por el contrario, en el presente estudio se encontró una asociación estadística del alelo T y el genotipo homocigoto de este polimorfismo con una disminución del cHDL en el grupo de casos.

En cuanto a las variables relacionadas con el metabolismo de la glucosa, Yang, *et al*., informaron que el polimorfismo rs1137101 del gen *LEPR* se asoció con cifras elevadas de HbA1C ^34^, y Boumaiza, *et al.*, demostraron su asociación con un aumento de glucemia basal e insulinemia en voluntarios de Túnez ^32^. Asimismo, Garcés, *et al.*, concluyeron en su estudio que el genotipo A/A de este polimorfismo confería a los niños obesos que lo portaban 2,6 veces más riesgo de desarrollar resistencia a la insulina que aquellos con los genotipos G/G y A/G ^35^. Además, se pudo demostrar que esta variante genética puede considerarse como un factor de riesgo significativo para la diabetes mellitus de tipo II en sujetos de Malasia ^36^.

Estos polimorfismos también se han asociado con la presión arterial, pero no se ha encontrado significación estadística en dicha relación en algunas poblaciones. Así, Fan, *et al*., no encontraron una asociación del polimorfismo rs2167270 del gen *LEP* en una población multiétnica de Malasia ^37^, así como tampoco se observó la influencia de rs17782313 del gen *MC4R* en la presión arterial de mujeres chinas ^34^. En contraste, el alelo A del polimorfismo rs1137101 del gen *LEPR* se asoció con una elevación de la presión arterial sistólica ^37^ en sujetos brasileros hipertensos, en quienes se evidenció que los valores fueron significativamente menores en los hombres portadores de genotipos con, al menos, una copia del alelo C de rs17782313 del gen *MC4R*^38^. En concordancia con estos resultados, el análisis genético del presente estudio demostró que el genotipo CC del polimorfismo *rs17782313* del gen *MC4R* se asoció con un aumento de la presión arterial sistólica en el grupo de obesos.

Aunque los mecanismos que vinculan la obesidad con la hipertensión no han sido completamente aclarados, se sabe que el aumento de la actividad simpática del sistema nervioso central contribuye a la elevación de la presión arterial en los sujetos obesos. La evidencia indica que la leptina y el sistema de melanocortina, incluidos los receptores 4 de la melanocortina (MC4R), juegan un papel clave en la relación de la obesidad con el aumento de la actividad del sistema nervioso central y la hipertensión, lo cual representa un objetivo importante para el tratamiento de la obesidad y sus consecuencias metabólicas y cardiovasculares ^39^.

Este trabajo se vio limitado por el tamaño de la muestra, por lo que se recomienda hacer estudios con una muestra más grande que permita un análisis estadístico con mayor poder.

En conclusión, los polimorfismos *rs2167270* del gen *LEP,* rs1137101 del gen *LEPR* y rs17782313 del gen *MC4R* no se asociaron con la obesidad en la población analizada. El polimorfismo rs17782313 del gen *MC4R* fue el único que se asoció con el aumento de la presión arterial sistólica y con la disminución del cHDL en sujetos con obesidad.
